# PATTERN OF HEALTH-RELATED BEHAVIORS AND RESOURCES AS PREDICTOR OF MEDICAL CARE AND MORTALITY RISK AMONG OLDER ADULTS

**DOI:** 10.1093/geroni/igz038.2551

**Published:** 2019-11-08

**Authors:** Dongmei Zuo, Merril D Silverstein

**Affiliations:** 1 Aging Studies Institute (ASI), Syracuse University, Syracuse, New York, United States; 2 Syracuse University, Syracuse, New York, United States

## Abstract

This study investigates the patterns and consequences of a wide range of health-related behaviors and resources that include health-compromising behaviors, health-promoting behaviors, preventive health behaviors, and health risks coping resources. We aim to identify the empirically-derived subgroups of individuals with unique profiles of health behaviors and resources to determine how subgroup membership predicts health outcomes and medical care utilization four years later. Data derived from 5,067 respondents in the 2010 and 2014 waves of the Health and Retirement Study. Latent class analysis was used to define classes based on 13 indicators in the 2010 wave, which also provided sociodemographic and health-related covariates. Outcomes were measured over 4 years. Six latent subgroups were identified: “Best Behavior/Resources”, “Low Social Support “, “Low Physical Activity”, “High Substance Abuse”, “Low Preventive Tests”, and “Low Governmental Health Insurance”. Compared with the “Best” group, older adults identified as “Low Physical Activity” and “High Substance Abuse” were found to have higher mortality risks and a lower likelihood of seeing doctors and less nursing home nights; older adults with the lowest level of receiving flu shots, cholesterol and cancer screen test (“Low Preventive Tests”) reported a less likelihood of seeing doctors; respondents in “Low Governmental Health Insurance” subgroup were associated with a lower likelihood of hospital stay and more nursing home nights. Results suggest that distinct groups of older individuals characterized by their health behaviors and resources provide a basis for identifying the high-risk segment of the older population for intervention.

